# Supplementary material for: Endopolyploidy Variation in Wild Barley Seeds across Environmental Gradients in Israel
Source: Genes (Basel). 2021 May 10;12(5):711. doi: 10.3390/genes12050711 (PMC8151103; doi:10.3390/genes12050711)
Supplement: Supplementary file 1 [file genes-12-00711-s001.zip › genes-1166060-Suppl-update.pdf]

genes

MDPI

Supplementary Materials

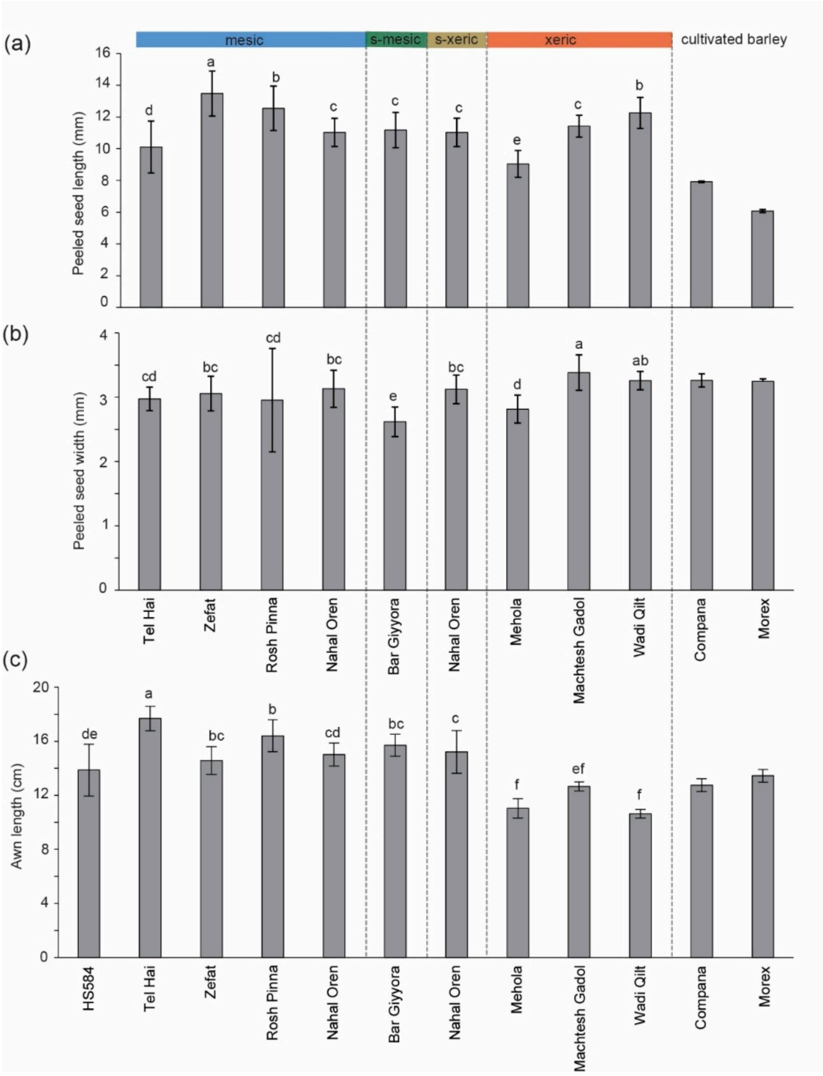

Figure S1. Phenotypic analysis of dry seeds of wild barley accessions originating from Israel. **Supports Figure 2. (a)** and **(b)** Quantitative data for peeled seed length and seed width. (**c**) Quantitative data for awn lengh. The ecological conditions for the sampling site of HS584 are unknown. s-mesic = semi-mesic, s-xeric = semi-xeric. Compana and Morex represent two- and six-rowed cultivated barley controls, respectively. Data are the means (±SD) from three biological repetitions, each with at least 20 seeds. One-way analysis of variance (ANOVA) was made separately for each tested parameter. Values marked with the same letter do not differ according to Duncan multiple range test ($p \leq 0.05$).

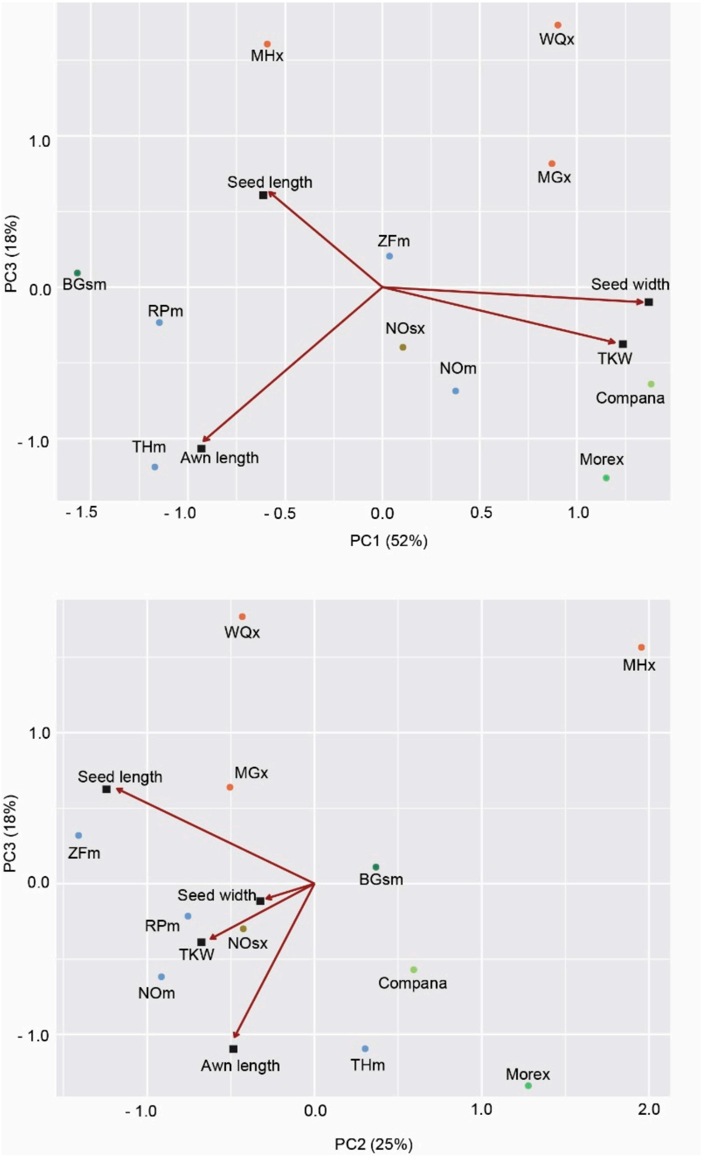

Figure S2. Principal component (PC) analysis of TKW, seed length and width for peeled seeds, and awn length. **Supports Figure 2.** The positions represent contribution rates of three PCs.

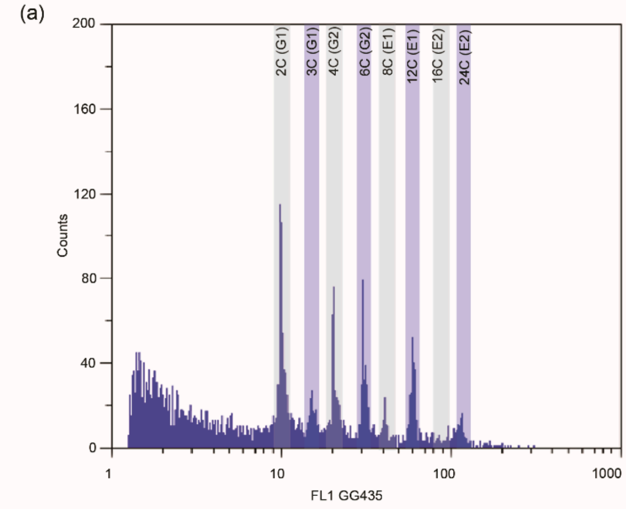

(b)

Gate	Count	%Gated
2C	960	57.5
4C	447	26.8
8C	200	12.0
16C	62	3.70
3C	380	23.17
6C	619	37.74
12C	407	24.82
24C	234	14.27

Figure S3. An example showing the interpretation of histograms of nuclear DNA content. **Supports Figures 3 and 4**. (**a**) Representative gated histogram obtained from 20 DAP whole peeled wild barley seed accession HS584. Histogram shows the gated C-value peaks for diploid seed tissue (a mixture of an embryo and seed maternal tissues; 2C = G1, 4C = G2, 8C = E1 = first endocycle, and 16C = E2 = second endocycle) and triploid endosperm tissues (3C = G1, 6C = G2, 12C = E1 = first endocycle, and 24C = E2= second endocycle) marked as gray and blue colored shading, respectively. The x-axis shows relative fluorescence intensity on log3 scale and y-axis number of measured particles (counts). The gates were created in FloMax program (Sysmex-Partec). The same gates were used for all evaluated samples of this study. (**b**) Table shows the number of particles per each gate collected from the histogram presented in (a).

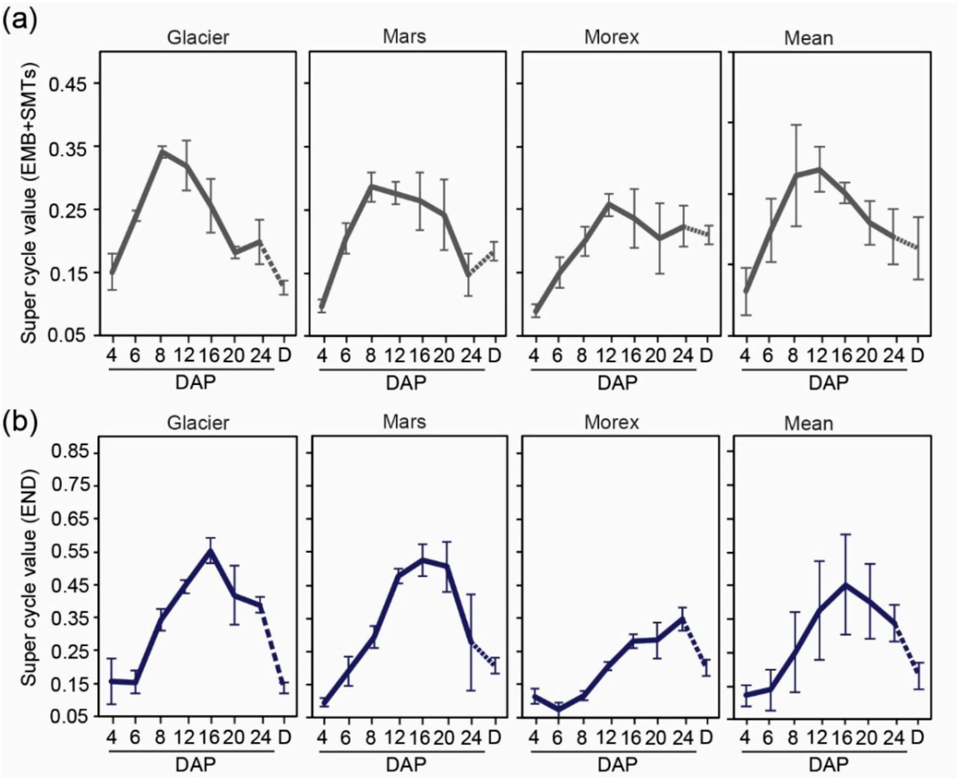

Figure S4. Comparison of super cycle values in seed tissues of three six-rowed barley cultivars. **Source data for Figure 4**. (**a**) and (**b**) show data for embryo/seed maternal tissues (EMB + SMTs) versus endosperm (END), respectively. Data are the means (±SD) from three biological replicates, each with at least five individual measurements. Mean = mean of super cycle value calculated between all cultivars. The dash-line between 24 DAP and dry seed samples means that seed development continued after 24 DAP.

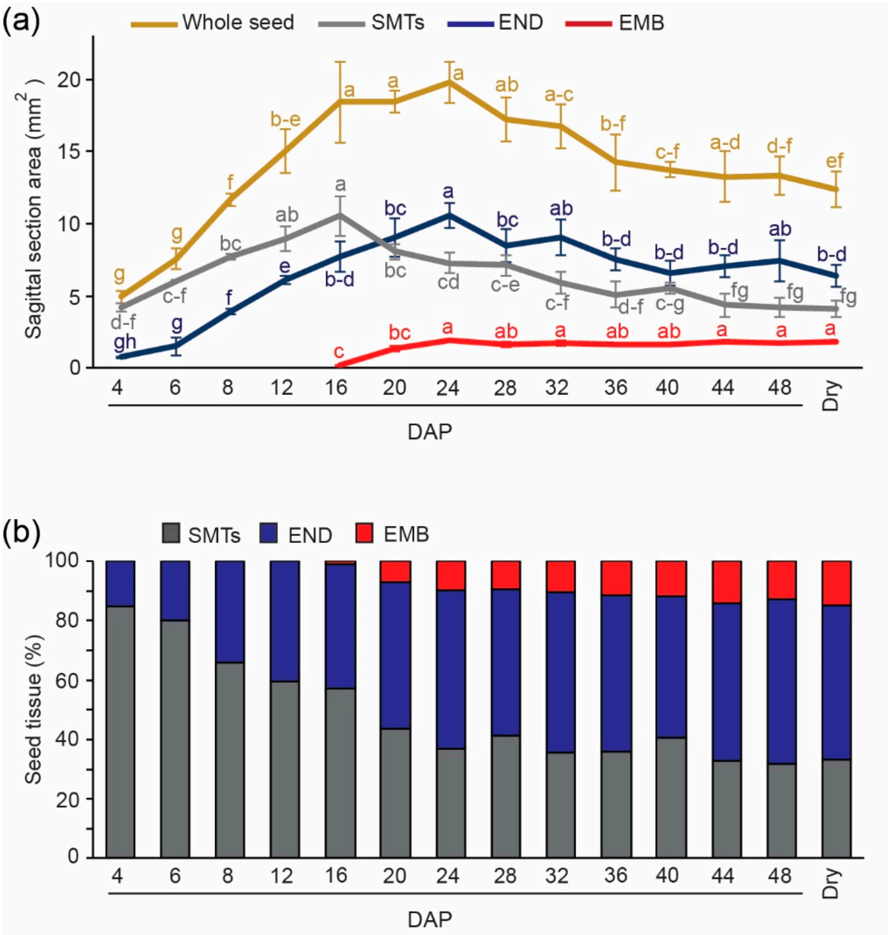

Figure S5. Time-course study of wild barley HS584 seed growth. **Supports Figure 6a.** (**a**) Measurements of sagittal section area of the whole seed, seed maternal tissues (SMTs), endosperm (END) and embryo (EMB) from 4 to 48 days after pollination (DAP) and in dry seed. Four to twelve DAP embryos were not analyzed for technical reasons. Data are the means from ≥20 individual measurements. ANOVA was made separately for each parameter. Values marked with the same letter do not differ according to Tukey's test ($p \leq 0.05$). (**b**) Cumulative percentage of major seed tissues calculated based on (a).

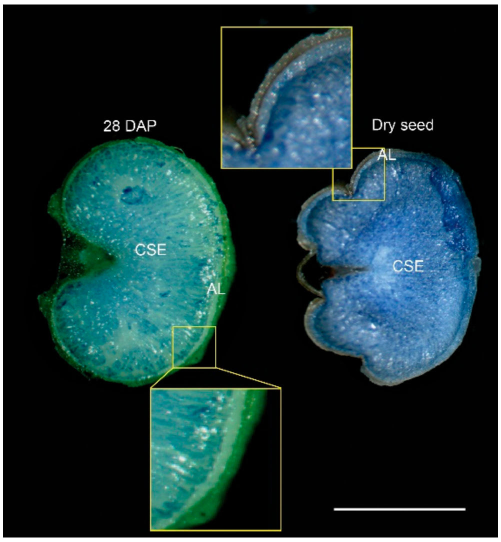

Figure S6. Analysis the aleurone layer viability. **Supports Figure 6b**. Selected transverse sections of 28 DAP and dry wild barley HS584 seeds stained with 0.1% Evans blue. AL = Aleurone layer, CSE = central starchy endosperm. Scale bar = 5 mm.

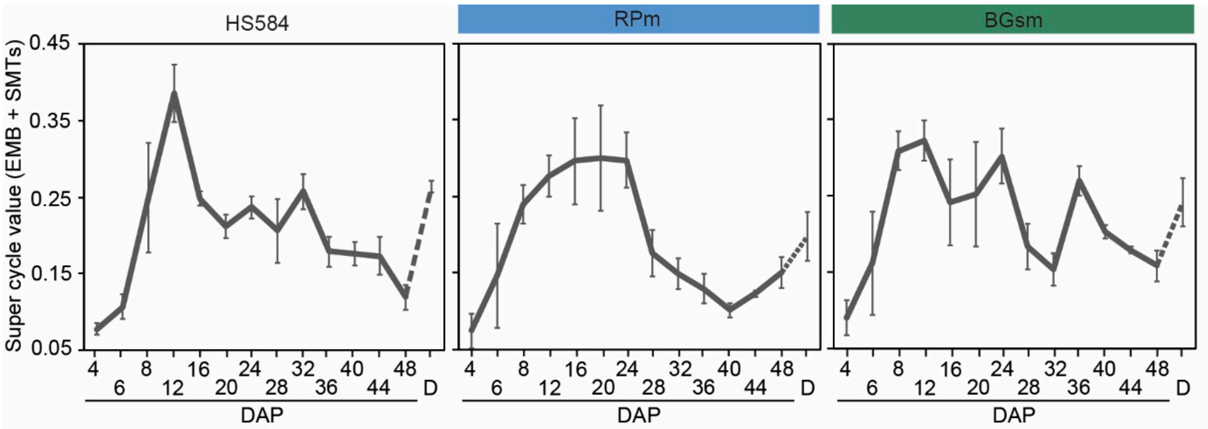

Figure S7. Comparison of super cycle values in diploid seed tissues (embryo + seed maternal tissues; EMB+SMTs) of three selected wild barley accessions at given DAP. D = Dry, RPm = Rosh Pinna, BGx = Bar Giyyora. **Complements Figure 6c**. Data are the means (±SD) from three biological replicates, each with at least five individual measurements. The dash-line between 48 DAP and dry seed samples means that seed desiccation continued after 48 DAP.

Tables S1. Percentage of 2C, 4C, 8C and 16C nuclei at a given DAP and in dry seeds in diploid seed tissues represented by the embryo (EMB) and seed maternal tissues (SMTs). **Supports Figure 3a.** Values are the means from 3 biological replicates, each with at least 5 measurements (seeds) ± SD. Blue color = mesic; green color = semi-mesic; green-brown color = semi-xeric; orange color = xeric.

4 DAP

	HS584	THm	ZFm	RPm	NOm	BGsm	NOsx	MHx	MGx	WQx
2C	42.9 ±2.98	42.1 ±1.85	48.7 ±0.16	45 ±2.4	48.2 ±0.72	43.6 ±2.86	47.5 ±1.8	45.8 ±0.79	51.5 ±0.23	49.4 ±0.54
4C	47.3 ±1.7	49.6 ±1.44	44.1 ±1.03	48.3 ±0.92	44.5 ±1.01	48 ±2.09	45.2 ±1.11	48.6 ±0.49	42.7 ±0.53	45.4 ±0.26
8C	9 ±1.39	7.6 ±1.3	6.7 ±0.92	5.9 ±1.85	6.6 ±0.81	7.7 ±0.89	6.5 ±0.63	4.9 ±1.11	5.2 ±0.26	4.7 ±0.29
16C	0.9 ±0.13	0.6 ±0.04	0.5 ±0.05	0.8 ±0.24	0.7 ±0.03	0.7 ±0.09	0.8 ±0.12	0.7 ±0.08	0.7 ±0.09	0.5 ±0.01

6 DAP

	HS584	THm	ZFm	RPm	NOm	BGsm	NOsx	MHx	MGx	WQx
2C	33.1 ±4.83	39 ±2.92	44.6 ±3.1	38.6 ±2.86	41.8 ±3.17	35.9 ±1.62	42.7 ±1.86	42.1 ±1.12	49.3 ±2.18	44.4 ±0.24
4C	45.6 ±0.82	48.9 ±1.89	44.3 ±1.06	48.7 ±2.07	43.7 ±1.84	49.3 ±1.02	47.2 ±1.35	48.9 ±0.42	42.4 ±1.47	45.8 ±0.87
8C	17.6 ±2.47	11 ±1.35	10.3 ±2.57	10.7 ±3.04	12.1 ±2.9	13.2 ±0.6	9.2 ±1.35	8 ±0.7	7.8 ±1.02	8.5 ±0.5
16C	3.7 ±2.37	1.1 ±0.25	0.8 ±0.07	2 ±1.89	2.4 ±2.17	1.5 ±0.26	0.9 ±0.09	1 ±0.19	0.5 ±0.11	1.4 ±0.48

8 DAP

	HS584	THm	ZFm	RPm	NOm	BGsm	NOsx	MHx	MGx	WQx
2C	30.1 ±2.49	35.9 ±2.82	37 ±3.64	33.6 ±2.08	42.6 ±0.58	33.8 ±1.01	37.7 ±1.62	40 ±2.03	35.9 ±0.97	42.8 ±2.31
4C	40.3 ±4.1	47.8 ±0.13	43.5 ±3.68	46.4 ±2.33	45.3 ±1.63	41.5 ±2.52	44.7 ±2.83	46 ±3.04	39.5 ±1.69	46.1 ±0.91
8C	20.7 ±2.15	14.6 ±2.62	16.4 ±4.17	15.9 ±0.99	10.5 ±1.01	18.6 ±1.44	13.7 ±2.59	10.5 ±0.85	20.7 ±1.13	10.1 ±2.29
16C	9 ±6.79	1.8 ±0.15	3.1 ±2.37	4 ±0.91	1.6 ±0.71	6.2 ±1.14	3.9 ±1.33	3.5 ±0.88	3.9 ±0.39	1 ±0.31

12 DAP

	HS584	THm	ZFm	RPm	NOm	BGsm	NOsx	MHx	MGx	WQx
2C	42.5 ±1.83	30.7 ±0.83	31.9 ±1.6	35.6 ±2.6	40.5 ±3.44	37.1 ±8.49	51 ±6.94	51.4 ±4.39	32.1 ±2.38	36.4 ±3
4C	34.1 ±1.29	41.8 ±0.75	36.9 ±3.64	42.3 ±2.48	37.2 ±2.15	37.2 ±5.77	33.2 ±7.05	32.4 ±1.98	38.3 ±0.51	34.9 ±2.16
8C	17.3 ±1.15	20.4 ±2.58	23.6 ±2.31	16.5 ±1.53	14 ±2.46	19.2 ±2.6	11.3 ±0.35	10.2 ±2.52	22.6 ±2.15	18 ±1.54
16C	6.2 ±0.84	7 ±1.3	7.5 ±1.64	5.5 ±1.59	8.2 ±1.25	6.5 ±0.2	4.5 ±1.01	6.1 ±0.41	7 ±0.66	10.6 ±3.89

16 DAP

	HS584	THm	ZFm	RPm	NOm	BGsm	NOsx	MHx	MGx	WQx
2C	51 ±0.19	38.5 ±7.08	50.9 ±2.39	45.8 ±3.28	50.6 ±1.61	51.8 ±11.07	51.4 ±7.86	51.9 ±2.2	40.8 ±5	45.8 ±6.76
4C	30.4 ±0.81	39 ±5.62	27 ±1	32 ±1.55	32.3 ±2.28	30.2 ±4.87	31.3 ±4.19	25.7 ±2.1	28.4 ±0.65	32 ±7.04
8C	11.9 ±0.71	16.1 ±3.46	13.2 ±1.72	14.7 ±1.41	10.6 ±1.93	11.8 ±4.96	11.9 ±2	11.2 ±0.87	20.1 ±3.51	14 ±2.02
16C	6.6 ±0.2	6.3 ±1.9	8.8 ±0.94	7.5 ±2.1	6.5 ±1.73	6.2 ±1.26	5.4 ±8.23	11.2 ±1	10.7 ±1.79	8.2 ±2.24

20 DAP

	HS584	THm	ZFm	RPm	NOm	BGsm	NOsx	MHx	MGx	WQx
2C	55.8 ±1.21	47 ±3.01	48.3 ±6	49.3 ±4.81	61.1 ±2.3	54.3 ±0.91	56.2 ±3	61.5 ±3.11	53.5 ±3.16	46.1 ±1.27
4C	28.5 ±2.07	35.5 ±3.1	28.7 ±0.56	29.1 ±2.06	27.1 ±2.27	27.9 ±0.64	29.2 ±1.14	23.9 ±1.51	28 ±0.99	25.9 ±1.29

8C	10.4 ±0.85	11.6 ±0.69	12.4 ±2.25	13.1 ±3.14	7.4 ±0.9	11.7 ±0.53	9.7 ±1.21	8.9 ±1.44	12.6 ±1.57	14.5 ±1.49
16C	5.3 ±0.47	6 ±0.93	10.5 ±4.35	8.5 ±1.87	4.4 ±0.66	6.1 ±0.79	4.9 ±0.66	5.8 ±0.61	5.9 ±0.93	13.5 ±0.96

24 DAP

	HS584	THm	ZFm	RPm	NOm	BGsm	NOsx	MHx	MGx	WQx
2C	53.2 ±1.52	47.9 ±3.04	49.3 ±3.86	51 ±1.41	60.8 ±4.82	51.2 ±2.23	57.3 ±1.72	57.1 ±3.94	48.4 ±4.82	47.8 ±1.41
4C	28.8 ±0.67	32.2 ±2.83	25.4 ±1.53	27.1 ±0.98	24.1 ±1.05	26.4 ±0.1	25.9 ±1.64	24.9 ±1.91	29.1 ±0.9	30.2 ±1.08
8C	12.3 ±0.73	12 ±1.57	13.8 ±1.41	14 ±0.73	9.8 ±2.91	14.5 ±1.43	11.3 ±1.15	11.2 ±0.94	15.1 ±3.48	15 ±0.71
16C	5.7 ±0.39	7.9 ±2.21	11.6 ±3.12	7.9 ±1.5	5.3 ±1.03	7.9 ±0.76	5.5 ±1.99	6.8 ±1.33	7.3 ±1.98	7 ±1.03

Dry

	HS584	THm	ZFm	RPm	NOm	BGsm	NOsx	MHx	MGx	WQx
2C	50 ±1.44	53.5 ±1.83	57 ±0.37	55.1 ±3.71	57.4 ±1.05	53.5 ±2.76	60.2 ±3.99	49.6 ±4.04	54.5 ±0.75	52.4 ±1.38
4C	28.4 ±0.97	28.3 ±1.15	28.2 ±0.75	28.7 ±1.27	28.9 ±1.32	27.4 ±2.23	27.6 ±1.34	29.7 ±2.32	28.4 ±0.57	27.8 ±0.79
8C	17 ±0.6	14.2 ±1.06	11.2 ±0.32	12.4 ±1.99	10.7 ±0.16	13.9 ±0.47	9.4 ±2.08	16.4 ±1.57	13.1 ±1	15.5 ±0.87
16C	4.7 ±0.31	4 ±0.3	3.6 ±0.2	3.8 ±0.49	3 ±0.3	5.2 ±0.33	2.8 ±0.73	4.4 ±0.18	3.9 ±0.15	4.3 ±0.31

Table S2. Super cycle values at a given DAP calculated for SMTs. **Supports Figure 3b.** Values are the means from 3 biological replicates, each with at least 5 measurements (seeds) ± SD.

	HS584	THm	ZFm	RPm	NOm	BGsm	NOsx	MHx	MGx	WQx
4 DAP	0.08 ± 0.01	0.09 ± 0.01	0.08 ± 0.01	0.07 ± 0.02	0.08 ± 0.01	0.09 ± 0.01	0.06 ± 0.01	0.07 ± 0.01	0.07 ± 0.00	0.06 ± 0.00
6 DAP	0.11 ± 0.02	0.13 ± 0.01	0.12 ± 0.03	0.15 ± 0.07	0.17 ± 0.07	0.16 ± 0.01	0.1 ± 0.01	0.1 ± 0.01	0.09 ± 0.01	0.11 ± 0.01
8 DAP	0.25 ± 0.07	0.18 ± 0.03	0.22 ± 0.07	0.24 ± 0.02	0.14 ± 0.02	0.31 ± 0.02	0.19 ± 0.01	0.18 ± 0.05	0.29 ± 0.02	0.12 ± 0.03
12 DAP	0.31 ± 0.04	0.35 ± 0.01	0.39 ± 0.03	0.28 ± 0.03	0.31 ± 0.01	0.32 ± 0.03	0.33 ± 0.03	0.22 ± 0.02	0.37 ± 0.03	0.39 ± 0.09
16 DAP	0.25 ± 0.01	0.29 ± 0	0.31 ± 0.02	0.3 ± 0.06	0.24 ± 0.05	0.24 ± 0.03	0.32 ± 0.07	0.23 ± 0.01	0.42 ± 0.07	0.3 ± 0.03
20 DAP	0.21 ± 0.02	0.24 ± 0.02	0.4 ± 0.01	0.3 ± 0.07	0.16 ± 0.02	0.25 ± 0.03	0.29 ± 0.01	0.2 ± 0.03	0.24 ± 0.03	0.42 ± 0.03
24 DAP	0.24 ± 0.01	0.28 ± 0.03	0.37 ± 0.05	0.3 ± 0.04	0.2 ± 0.05	0.3 ± 0.04	0.28 ± 0.03	0.25 ± 0.05	0.3 ± 0.07	0.29 ± 0.03
Dry	0.26 ± 0.01	0.22 ± 0.01	0.18 ± 0.01	0.2 ± 0.03	0.17 ± 0.01	0.24 ± 0.02	0.15 ± 0.01	0.25 ± 0.03	0.26 ± 0.02	0.24 ± 0.01

	mesic	semi-mesic	semi-xeric	xeric
4 DAP	0.08 ± 0.01	0.09 ± 0.01	0.06 ± 0.01	0.06 ± 0.01
6 DAP	0.14 ± 0.02	0.16 ± 0.01	0.1 ± 0.01	0.1 ± 0.01
8 DAP	0.19 ± 0.04	0.31 ± 0.02	0.19 ± 0.01	0.19 ± 0.08
12 DAP	0.33 ± 0.05	0.32 ± 0.03	0.33 ± 0.03	0.33 ± 0.09
16 DAP	0.28 ± 0.03	0.24 ± 0.03	0.32 ± 0.07	0.32 ± 0.09
20 DAP	0.27 ± 0.1	0.25 ± 0.03	0.29 ± 0.01	0.29 ± 0.11
24 DAP	0.29 ± 0.07	0.3 ± 0.04	0.28 ± 0.03	0.28 ± 0.03
Dry	0.19 ± 0.02	0.24 ± 0.02	0.15 ± 0.01	0.25 ± 0.01

Table S3. Percentage of 3C, 6C, 12C and 24C nuclei at a given DAP and in dry seeds in endosperm **Supports Figure 4a.** Values are the means from 3 biological replicates, each with at least 5 measurements (seeds) ± SD. Blue color = mesic; green color = semi-mesic; green-brown color = semi-xeric; orange color = xeric.

4 DAP

	HS584	THm	ZFm	RPm	NOm	BGsm	NOsx	MHx	MGx	WQx
3C	58.9 ±7.36	60 ±6.66	62.4 ±8.92	62.6 ±4.73	51 ±13.62	59.1 ±1.25	59.3 ±6.22	50.5 ±1.36	80.2 ±2.12	66.6 ±1.87
6C	32.5 ±6.96	32 ±7.01	30.4 ±9.12	29.6 ±6.05	39.4 ±14.71	31.5 ±1.61	33.1 ±6.04	40 ±3.74	14.3 ±1.21	27.2 ±1.94
12C	6.7 ±0.86	6.4 ±0.3	5.6 ±1.05	6.1 ±1.97	7.4 ±0.42	7.4 ±1.97	5.9 ±0.54	7 ±2.41	3.9 ±0.64	4.8 ±0.62
24C	1.8 ±0.19	1.4 ±0.33	1.6 ±0.46	1.6 ±0.94	2.1 ±0.6	1.9 ±0.84	1.7 ±0.15	2.4 ±0.72	1.4 ±0.22	1.2 ±0.07

6 DAP

	HS584	THm	ZFm	RPm	NOm	BGsm	NOsx	MHx	MGx	WQx
3C	50.9 ±4.4	59.7 ±3.1	55.3 ±9.61	55.5 ±7.19	56.1 ±6.98	53.4 ±0.61	61.6 ±6.59	50.2 ±5.67	75.3 ±3.1	56.9 ±1.12
6C	34 ±3.47	34.1 ±1.58	35.6 ±6.87	33.7 ±0.93	30.2 ±2.91	36.1 ±1.7	31.7 ±6.31	40.4 ±4.69	19.8 ±3.25	35.1 ±0.91
12C	12.7 ±6.18	5 ±1.08	7.1 ±2.18	8.8 ±5.87	10.2 ±6.14	9 ±1.67	5.4 ±0.95	7.6 ±1.2	3.9 ±0.32	6.6 ±1.33
24C	2.4 ±1.71	1.1 ±0.45	1.9 ±0.73	1.9 ±0.82	3.3 ±3.29	1.5 ±0.45	1.1 ±0.02	1.6 ±0.33	1 ±0.21	1.3 ±0.10

8 DAP

	HS584	THm	ZFm	RPm	NOm	BGsm	NOsx	MHx	MGx	WQx
3C	48.3 ±1.35	54.9 ±2.7	49.8 ±9.33	47.9 ±3.15	56.2 ±6.73	39.6 ±1.55	52.2 ±3.93	44.5 ±3.35	47 ±2.17	61.2 ±1.19
6C	27.5 ±3.79	35.2 ±2.32	32.2 ±1.79	34.5 ±3.82	29.8 ±2.82	30.3 ±1.83	30 ±3.09	30.5 ±0.73	32.8 ±1.38	33.2 ±1.5
12C	18.4 ±0.45	8.1 ±0.69	14.1 ±6.61	15.1 ±0.2	10 ±3.09	23 ±1.1	14.7 ±3.08	17.9 ±2.11	17.1 ±2.09	4.6 ±0.7
24C	5.8 ±2.12	1.7 ±0.53	3.9 ±3.49	2.3 ±0.5	2.9 ±1.9	7 ±0.96	3.1 ±3.79	7 ±1.08	3 ±0.48	0.9 ±0.2

12 DAP

	HS584	THm	ZFm	RPm	NOm	BGsm	NOsx	MHx	MGx	WQx
3C	27.1 ±5.35	33.2 ±7.78	35.8 ±6.77	44.4 ±4.04	35.8 ±0.82	37.4 ±7.42	33.4 ±2.99	22 ±4.27	40.5 ±10.28	26.5 ±6.08
6C	34.3 ±2.63	34.5 ±1.69	32.3 ±0.99	32 ±1.79	31.1 ±0.94	29.9 ±0.36	31 ±1.19	30.3 ±0.76	31.4 ±3.61	39.4 ±1.62
12C	26.4 ±3.4	24.3 ±4.63	22.2 ±3.28	19 ±2.11	24.3 ±1.21	21.1 ±4.64	25.2 ±1.38	29.1 ±0.79	21.3 ±5.9	28.6 ±3.76
24C	11.9 ±0.55	7.9 ±1.51	9.5 ±3.92	4.5 ±1.98	8.7 ±0.49	11.2 ±2.57	10.3 ±0.71	18.3 ±4.08	6.6 ±1.22	5.4 ±0.79

16 DAP

	HS584	THm	ZFm	RPm	NOm	BGsm	NOsx	MHx	MGx	WQx
3C	25.7 ±1.98	35 ±15.36	18.4 ±1.84	22.9 ±1.69	27.1 ±1.89	22.5 ±5.45	28.8 ±2.09	21.1 ±2.3	17.4 ±2.77	30.9 ±12.86
6C	34.1 ±0.65	33.5 ±3.36	33.2 ±3.11	42.5 ±1.46	30.9 ±1.56	32.1 ±1.31	30.1 ±4.78	25.2 ±2.83	37.3 ±1.06	36.6 ±1.9
12C	29.1 ±1.58	23.3 ±8.89	34.4 ±1.76	28.4 ±1.38	29.8 ±0.22	32.9 ±3.56	28 ±4.29	29.6 ±0.79	29.8 ±1.83	26.1 ±7.69
24C	10.8 ±2.74	7.7 ±4.07	13.6 ±2.77	6.2 ±1.57	11.8 ±0.51	12.3 ±0.61	12.9 ±6.16	23.5 ±5.29	15 ±1.98	6.3 ±3.33

20 DAP

	HS584	THm	ZFm	RPm	NOm	BGsm	NOsx	MHx	MGx	WQx
3C	29 ±3.01	27.8 ±2.04	21.4 ±2.05	22.7 ±3.27	26.8 ±3.3	24.1 ±1	26.6 ±2.94	24.2 ±3.17	21.4 ±2.54	17.6 ±1.29
6C	37.6 ±2.43	36.1 ±0.67	33.7 ±0.94	38.7 ±0.42	29.4 ±2.13	30.4 ±1.78	32.7 ±1.3	31 ±1.64	32 ±0.64	31.2 ±2.66
12C	25.5 ±1.6	26.3 ±2.67	30.9 ±1	31 ±2.39	32.2 ±2.36	31.9 ±0.17	30.7 ±2.39	26.9 ±7.75	33.9 ±2.28	36.6 ±1.9
24C	7.7 ±1.03	9.5 ±1.55	13.4 ±1.46	7.5 ±1.5	11.4 ±1.13	13.2 ±2.95	9.9 ±3.26	17.7 ±3.01	12.3 ±0.89	14.1 ±2.19

24 DAP

	HS584	THm	ZFm	RPm	NOm	BGsm	NOsx	MHx	MGx	WQx
3C	26.2 ±0.58	26.9 ±1.4	25.7 ±3.01	22.7 ±1.53	28.2 ±2.34	22.8 ±2.1	30.1 ±2.19	31.9 ±2.54	20 ±2.04	23.8 ±2.11
6C	34.2 ±0.71	33.8 ±1.8	29.1 ±2.47	38 ±3.95	29.4 ±2.79	30.7 ±1.82	30.8 ±2.65	33 ±0.28	32 ±1.82	35.2 ±3.26
12C	29.1 ±0.98	31.2 ±1.12	28.9 ±1.74	31.8 ±2.43	30.7 ±1.87	32.7 ±1.65	28.7 ±2.55	23 ±5.92	34.6 ±0.46	31 ±2.2
24C	10.5 ±0.9	8 ±1.61	15.8 ±2.84	7.4 ±3.42	11.6 ±1.72	13.6 ±1.43	10.3 ±0.78	11.8 ±3.46	13.1 ±4.03	9.7 ±3.22

Dry

	HS584	THm	ZFm	RPm	NOm	BGsm	NOsx	MHx	MGx	WQx
3C	31.1 ±1.13	37.2 ±2.29	38.7 ±1.81	39.5 ±1.31	46.8 ±1.38	38.1 ±2.27	44.9 ±1.53	28.9 ±3.77	38.2 ±2.25	37 ±1.37
6C	51.8 ±2.33	47.2 ±2.5	41.2 ±1.7	45.8 ±3.2	39.5 ±2.09	39.5 ±4.21	36.8 ±2.36	57 ±2.08	44.9 ±1.03	46.5 ±1.96
12C	12.5 ±0.82	12 ±0.24	15.1 ±0.88	11.3 ±1.46	10.5 ±0.29	15.5 ±2.27	13.4 ±1.64	11.4 ±1.35	12.7 ±3.26	12.3 ±0.81
24C	4.2 ±1.65	3.5 ±0.2	4.8 ±0.51	3.3 ±0.46	3.1 ±0.52	6.6 ±0.4	4.5 ±0.53	2.6 ±0.49	4.1 ±3.07	4 ±0.15

Table S4. Super cycle values at a given DAP calculated for endosperm. **Supports Figure 4b.** Values are the means from 3 biological replicates, each with at least 5 measurements (seeds) ± SD.

	HS584	THm	ZFm	RPm	NOm	BGsm	NOsx	MHx	MGx	WQx
4 DAP	0.16 ± 0.04	0.1 ± 0.01	0.09 ± 0.02	0.09 ± 0.04	0.12 ± 0.02	0.12 ± 0.04	0.09 ± 0.04	0.11 ± 0.01	0.07 ± 0.01	0.08 ± 0.01
6 DAP	0.11 ± 0.01	0.07 ± 0.02	0.11 ± 0.03	0.13 ± 0.07	0.1 ± 0	0.12 ± 0.01	0.08 ± 0.03	0.11 ± 0.01	0.06 ± 0.01	0.1 ± 0.01
8 DAP	0.18 ± 0.08	0.12 ± 0.01	0.22 ± 0.11	0.2 ± 0.01	0.19 ± 0.1	0.38 ± 0.04	0.2 ± 0.02	0.32 ± 0.11	0.23 ± 0.03	0.07 ± 0.01
12 DAP	0.3 ± 0.04	0.4 ± 0.08	0.42 ± 0.11	0.28 ± 0.06	0.42 ± 0.02	0.45 ± 0.08	0.46 ± 0.1	0.67 ± 0.02	0.35 ± 0.08	0.4 ± 0.05
16 DAP	0.53 ± 0.05	0.5 ± 0.03	0.63 ± 0.07	0.41 ± 0.03	0.55 ± 0.01	0.58 ± 0.1	0.54 ± 0.05	0.78 ± 0.1	0.61 ± 0.04	0.47 ± 0.02
20 DAP	0.42 ± 0.02	0.46 ± 0.03	0.59 ± 0.04	0.47 ± 0.05	0.55 ± 0.04	0.6 ± 0.02	0.51 ± 0.06	0.63 ± 0.06	0.6 ± 0.03	0.66 ± 0.04
24 DAP	0.5 ± 0.01	0.48 ± 0.03	0.62 ± 0.07	0.47 ± 0.09	0.54 ± 0.03	0.6 ± 0.02	0.5 ± 0.04	0.47 ± 0.01	0.61 ± 0.08	0.51 ± 0.05
Dry	0.22 ± 0.04	0.19 ± 0	0.18 ± 0.02	0.18 ± 0.02	0.17 ± 0.01	0.3 ± 0.02	0.19 ± 0.02	0.17 ± 0.03	0.22 ± 0.04	0.21 ± 0

	mesic	semi-mesic	semi-xeric	xeric
4 DAP	0.1 ± 0.01	0.12 ± 0.01	0.09 ± 0.01	0.09 ± 0.02
6 DAP	0.1 ± 0.02	0.12 ± 0.01	0.08 ± 0.01	0.09 ± 0.03
8 DAP	0.18 ± 0.04	0.38 ± 0.02	0.2 ± 0.01	0.21 ± 0.13
12 DAP	0.38 ± 0.07	0.45 ± 0.03	0.46 ± 0.03	0.47 ± 0.17
16 DAP	0.52 ± 0.09	0.58 ± 0.03	0.54 ± 0.07	0.62 ± 0.15
20 DAP	0.52 ± 0.06	0.6 ± 0.03	0.51 ± 0.01	0.63 ± 0.03
24 DAP	0.53 ± 0.07	0.6 ± 0.04	0.5 ± 0.03	0.53 ± 0.07
Dry	0.18 ± 0.01	0.3 ± 0.02	0.19 ± 0.01	0.2 ± 0.03

Table S5. Super cycle values at a given DAP calculated for embryo/seed maternal tissues (EMB+SMTs) and endosperm (END). Data are the means (±SD) from three biological replicates, each with at least 5 individual measurements. **Supports Figure 5a**.

EMB + SMTs	Wild	Cultivated
4 DAP	0.07 ± 0.01	0.11 ± 0.03
6 DAP	0.12 ± 0.03	0.2 ± 0.04

END	Wild	Cultivated
4 DAP	0.1 ± 0.02	0.11 ± 0.02
6 DAP	0.1 ± 0.02	0.14 ± 0.05

8 DAP	0.21 ± 0.06	0.29 ± 0.05	8 DAP	0.21 ± 0.09	0.26 ± 0.08
12 DAP	0.33 ± 0.05	0.32 ± 0.06	12 DAP	0.41 ± 0.11	0.39 ± 0.11
16 DAP	0.29 ± 0.06	0.26 ± 0.04	16 DAP	0.56 ± 0.1	0.46 ± 0.13
20 DAP	0.27 ± 0.08	0.21 ± 0.04	20 DAP	0.55 ± 0.08	0.43 ± 0.1
24 DAP	0.28 ± 0.04	0.18 ± 0.03	24 DAP	0.53 ± 0.06	0.36 ± 0.07
Dry	0.22 ± 0.04	0.17 ± 0.03	Dry	0.2 ± 0.04	0.19 ± 0.02
